# *Notes from the Field:* First Locally Acquired Dengue Virus Infections — Pasadena, California, October–December 2023

**DOI:** 10.15585/mmw.mm7342a4

**Published:** 2024-10-24

**Authors:** Matt Feaster, Rudy Patrick, Michael Oshiro, Melody Kuan, Ying-Ying Goh, Manuel Carmona, Sara Y. Tartof, Jason Farned, Tristan Hallum, Andrea J. Lund, Chris Preas, Sharon Messenger, Vicki Kramer, Mary Danforth, Colten Sheridan

**Affiliations:** ^1^Pasadena Public Health Department, Pasadena, California; ^2^Epidemic Intelligence Service, CDC; ^3^Department of Research & Evaluation, Kaiser Permanente Southern California, Pasadena, California; ^4^San Gabriel Valley Mosquito and Vector Control District, West Covina, California; ^5^California Department of Public Health.

**Summary**

**What is already known about this topic?**

During 2013–2022, six cases of travel-acquired dengue were reported in Pasadena, California.

**What is added by this report?**

On October 2, 2023, the Pasadena Public Health Department received a laboratory report of elevated dengue antibodies from a symptomatic patient with no recent travel history. Dengue virus 1 infection was confirmed on October 18 by reverse transcription–polymerase chain reaction. Subsequent epidemiologic investigation of the surrounding neighborhood identified an asymptomatic second person with infection. Vector mitigation, conducted during October 2–9, resulted in a 62% reduction of trapped adult mosquitoes.

**What are the implications for public health practice?**

Swift vector mitigation response reduced adult mosquito population levels to lower community risk. Partnerships among local health departments and vector control agencies are important to ensure rapid response to locally acquired dengue cases.

Global incidence of dengue is increasing (*1*). Dengue, a mosquitoborne arboviral disease caused by four dengue viruses (DENV 1–4), is transmitted to humans by *Aedes* species mosquitoes; invasive *Aedes *species are found in California (*2*,*3*). During 2013–2022, six cases of travel-acquired dengue were reported in Pasadena, California. On October 2, 2023, the Pasadena Public Health Department (PPHD) received a laboratory report of elevated dengue antibodies in a symptomatic patient with no recent travel history. The most common symptom of dengue is fever and can include headache; pain behind the eyes; muscle, joint, or bone pain; nausea; vomiting; and rash (*2*). The patient (person A) first experienced symptoms of arboviral illness in mid-September 2023, and required hospitalization. PPHD activated community risk mitigation measures and conducted an epidemiologic investigation in coordination with the San Gabriel Valley Mosquito and Vector Control District (SGVMVCD).

## Investigations and Outcomes

### Vector Risk Mitigation

On October 2, PPHD alerted SGVMVCD that person A had a suspected case of arboviral disease. During October 2–9, SGVMVCD trapped, counted, and tested mosquitoes within 0.16 miles (250 m) of person A’s home, per guidance from the California Department of Public Health* (*4*). *Aedes* mosquito counts were eight times as high as in other routine surveillance areas in the San Gabriel Valley for the same period (80 versus an average of 10.5 adult *Aedes* mosquitoes per trap) (*5*). During the same period, SGVMVCD conducted a door-to-door mosquito prevention educational campaign and two rounds of truck-mounted adulticide and larvicide treatments in the San Gabriel Valley. Mosquito trap counts 1 week after baseline confirmed a 62% reduction in the mean number of trapped adult mosquitoes (80 per trap pretreatment versus 30 posttreatment). Reverse transcription–polymerase chain reaction (RT-PCR) testing on mosquito pools were negative for arboviruses.

### Public Health Investigations

PPHD activated community risk mitigation measures and conducted an epidemiologic investigation. This activity was reviewed by CDC, deemed not research, and was conducted consistent with applicable federal law and CDC policy.^†^

Interviews with person A confirmed no recent travel history. The objectives of the investigation were to identify a potential index case preceding person A with relevant travel, identify potential additional secondary infections, and conduct enhanced case investigation in the surrounding neighborhood. During October 10–November 14, PPHD attempted to contact 175 households; 14 (8.0%) declined, and 31 (17.7%) could not be reached. Among the 130 (74.3%) households with a completed interview, 15 (12%) had a member with recent travel to regions where dengue is endemic, eight (6%) had a member with recent symptoms consistent with arboviral illness, and one (1%) had a member with both. Testing by RT-PCR confirmed person A to have DENV-1 infection on October 18. On December 5, follow-up interviews of the 24 households with at least one person with travel or symptoms were conducted, and six consenting adults provided serum samples. From these samples, one additional person (person B) was confirmed as having DENV-1 infection by RT-PCR. Person B had no recent travel history, no symptoms consistent with dengue, and no reported contact with recent travelers or symptomatic persons. 

## Preliminary Conclusions and Actions

This first confirmed symptomatic case of locally acquired dengue in California was identified in October 2023, with subsequent evidence of a second asymptomatic person with infection. Rapid deployment of vector control resources and epidemiologic investigations before laboratory confirmation were vital to timely mitigation of arboviral risk. Established, active partnership among public health and vector control agencies is important for rapid reduction of mosquito populations when local transmission of arboviral disease is suspected.

## Footnotes

* This information was current when accessed October 2, 2023. In 2023, the recommended radius was 0.16 miles (250 meters); however, in 2024, the recommended radius is 0.09 miles (150 meters).

† 45 C.F.R. part 46.102(l)(2), 21 C.F.R. part 56; 42 U.S.C. Sect. 241(d); 5 U.S.C. Sect. 552a; 44 U.S.C. Sect. 3501 et seq.
